# Static and Fatigue Mechanical Performance of Abutments Materials for Dental Restorations

**DOI:** 10.3390/ma16103713

**Published:** 2023-05-13

**Authors:** Luigi Bruno, Luigi Canullo, Yaniv Mayer, Todd Schoenbaum, Francesco Giuzio, Carmine Maletta

**Affiliations:** 1 Department of Mechanical Energy and Management Engineering, University of Calabria, 87036 Rende, Italy; 2 Department of Surgical Sciences, University of Genoa, 16132 Genova, Italy; 3 Department of Periodontology, School of Post Graduate Dentistry, Rambam Health Care Campus, Haifa P.O. Box 9602, Israel; 4 School of Dentistry, University of California-Los Angeles, Los Angeles, CA 90095, USA; 5 Dental Medicine and Oral Surgery, 87100 Cosenza, Italy

**Keywords:** mechanical characterization, static strength, fatigue strength, abutment, dental implant

## Abstract

The choice of the proper restorative material is essential for the long-term success of implant-supported rehabilitations. This study aimed to analyze and compare the mechanical properties of four different types of commercial abutment materials for implant-supported restorations. These materials included: lithium disilicate (A), translucent zirconia (B), fiber-reinforced polymethyl methacrylate (PMMA) (C), and ceramic-reinforced polyether ether ketone (PEEK) (D). Tests were carried out under combined bending–compression conditions, which involved applying a compressive force tilted with respect to the abutment axis. Static and fatigue tests were performed on two different geometries for each material, and the results were analyzed according to ISO standard 14801:2016. Monotonic loads were applied to measure static strength, whereas alternating loads with a frequency of 10 Hz and a runout of 5 × 10^6^ cycles were applied for fatigue life estimation, corresponding to five years of clinical service. Fatigue tests were carried out with a load ratio of 0.1 and at least four load levels for each material, and the peak value of the load levels was reduced accordingly in subsequent levels. The results showed that the static and fatigue strengths of Type A and Type B materials were better than those of Type C and Type D. Moreover, the fiber-reinforced polymer material, Type C, showed marked material–geometry coupling. The study revealed that the final properties of the restoration depended on manufacturing techniques and the operator’s experience. The findings of this study can be used to inform clinicians’ choice of restorative materials for implant-supported rehabilitation, considering factors such as esthetics, mechanical properties, and cost.

## 1. Introduction

The choice of the proper restorative material is fundamental for the long-term success of implant-supported rehabilitation [1]. Several synthetic materials are available nowadays for manufacturing implant crowns and bridges, including metals, ceramics, polymers, and composites [1]. Different factors, including esthetics, mechanical properties, and the cost might affect the clinicians’ choice [1,2]. The systematic review and metanalysis by Pjetursson et al. [2] demonstrated that the incidence of technical complications is significantly higher for implant-supported reconstructions compared to that of tooth-supported fixed partial dentures [3]. It is possible that the absence of a periodontal ligament, which typically provides cushioning against occlusal loads in natural teeth, may contribute to this phenomenon. As a result, some authors have proposed the utilization of resinous materials for implant-supported restorations [4]. While ceramics are the most esthetic restorative materials and are characterized by a very high degree of stiffness, resin and composite resins are more elastic and exhibit a higher shock absorption capacity. In vitro studies demonstrated that they are able to dampen occlusal loads [4,5,6]. This could be of significant importance, especially in full-arch, immediate loading restorations, where dental implants are loaded prior to achieving osseointegration. Therefore, it is crucial to exert the maximum amount of effort in controlling occlusal loads to minimize the risk of implant micromovement [7]. Polymers have evolved a lot in recent decades, and they might be used both as a veneering material and as a framework material, mainly depending on the type of filler. In particular, fiber-reinforced composites have demonstrated mechanical properties similar to those of metal alloys, and they have been proposed for the realization of substructures of implant-supported restorations [8,9,10,11,12]. The advantages of polymers widely used for composites [13,14,15] include that they are easy to repair, lightweight, and cheap. Although they possess low abrasion coefficients, it is important to emphasize that the final properties of the restoration are heavily reliant on the manufacturing techniques employed, and the outcomes may be influenced by the operator’s experience. Conversely, zirconia and ceramics have emerged as the predominant materials for implant prosthodontics [2,16]. They are rigid, inorganic materials, with a high coefficient of abrasion and good esthetic properties. The most common complications are fractures or the chipping of the veneering material. Technical complications are quite common in implant-supported restorations and might strongly affect the patients’ comfort and satisfaction with their oral rehabilitation. Therefore, it is crucial to thoroughly examine the mechanical properties of restorative materials intended for use in implant dentistry, particularly when they are subjected to cyclic loading conditions [17,18,19].

Within this context, in the present study, we aimed to analyze the mechanical performance of four different types of commercial abutment materials, both ceramics and polymer composites. In particular, the static and fatigue strength of abutments obtained from the selected materials were analyzed and systematically compared according to the ISO standard 14801:2016 [20]. Two different geometries for each material were analyzed, but just for one of them, the Student’s *t*-test was applied to the static strength results, revealed the significant influence of specimen shape on the mechanical resistance of the abutment. The results attained in the static tests guided the fatigue characterization campaign that was carried out with the aim of simulating five years of clinical service, corresponding to 5 × 10^6^ loading cycles [17,21]. The fatigue tests conducted on each material and/or geometry produced the power law fatigue curve and related parameters, with which it was possible to evaluate the different sensitivities to the fatigue loading conditions.

## 2. Materials and Methods

The experimental tests were carried out according to ISO standard 14801:2016 [20], which specifies a method for the dynamic testing of a single dental implant of the transmucosal type in combination with its prosthetic components. The standard was largely designed to compare implants characterized by different designs, sizes, and materials; although, the fatigue life of a specimen with a specific geometry can be evaluated as well.

### 2.1. Experimental Apparatus

The testing machine used to perform the fatigue tests was the Instron Electropuls^®^ E10000 [2], which consists of a linear-torsion, all-electric dynamic test instrument. This machine is based on decoupled linear/rotary actuators with a ±10 kN dynamic linear load capacity and a ±100 Nm dynamic torque load capacity capable of producing above 100 Hz. Both load cells have the ability to measure forces and torques as low as 1/250th of their capacities, with an accuracy of 0.5%. Additionally, 60 mm linear strokes and 16-revolution rotations give the machine a high level of versatility to analyze diverse types of specimens and materials.

**Figure 1:**
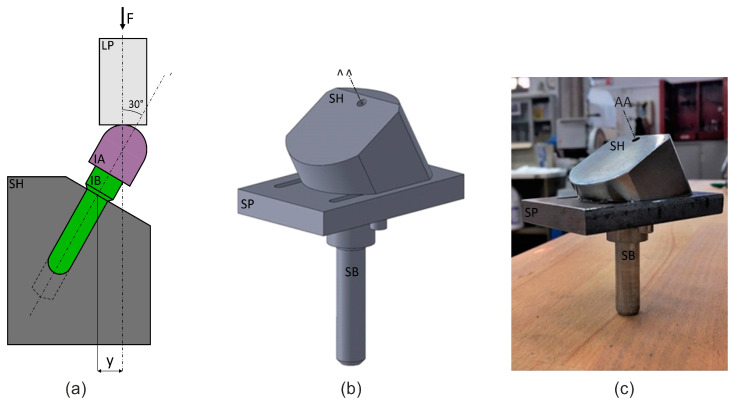
Loading geometry and setup: (**a**) a schematic of the whole system, including specimen holder, specimen, and loading punch; (**b**) a virtual model of the same system and (**c**) the actual assembly, including the specimen holder, a plate, and a bar that served to fix the specimen to the bottom (static) crossbar. In figure: F, the axial load; LP, the loading punch; IA, the implant abutment; IB, the implant body; SH, the specimen holder; y, the moment arm; SP, the support plate; AA, abutment axis; SB, the support bar.

The standard defines two possible loading geometries for systems with or without angulated abutments. In the present work, a system with no angulated abutment was adopted, the schematic of which is shown (see in Figure 1a). Load F was applied with the load punch, LP, which was fixed in the upper (movable) crossbar. The specimen comprised of the implant abutment, IA, and the implant body, IB, which were attached to each other through a cementation process, was fixed in a blind hole drilled into the specimen holder, SH. The quantity y is the arm used to calculate the bending moment acting on the implant.

The loading setup was configured according to this load geometry. Autodesk Inventor 2022 (education version) CAD software was used to model the system (Figure 1b) in order to define the correct shape and dimensions that would fit within the testing volume of the fatigue machine and fulfil the functions required of the loading setup in terms of stiffness and load transfer. The specimen holder, SH, was fixed with two screws onto the support plate, SP, on which two slots were milled to allow the translation necessary for specimen alignment. The support plate was fixed to the grip of the bottom (static) crossbar by way of the support bar, SB, which was screwed into the plate after threading one of its ends and through a hole in the plate itself.

As static and dynamic tests necessitated only a small load, there was no need to conduct numerical simulations in order to verify the stiffness or capacity of the loading setup. Figure 1c demonstrates the bottom part of the machined and assembled loading setup as it was placed in the testing machine. The loading punch consists of a steel rod fixed to the upper (movable) crossbar.

### 2.2. Specimen

**Figure 2:**
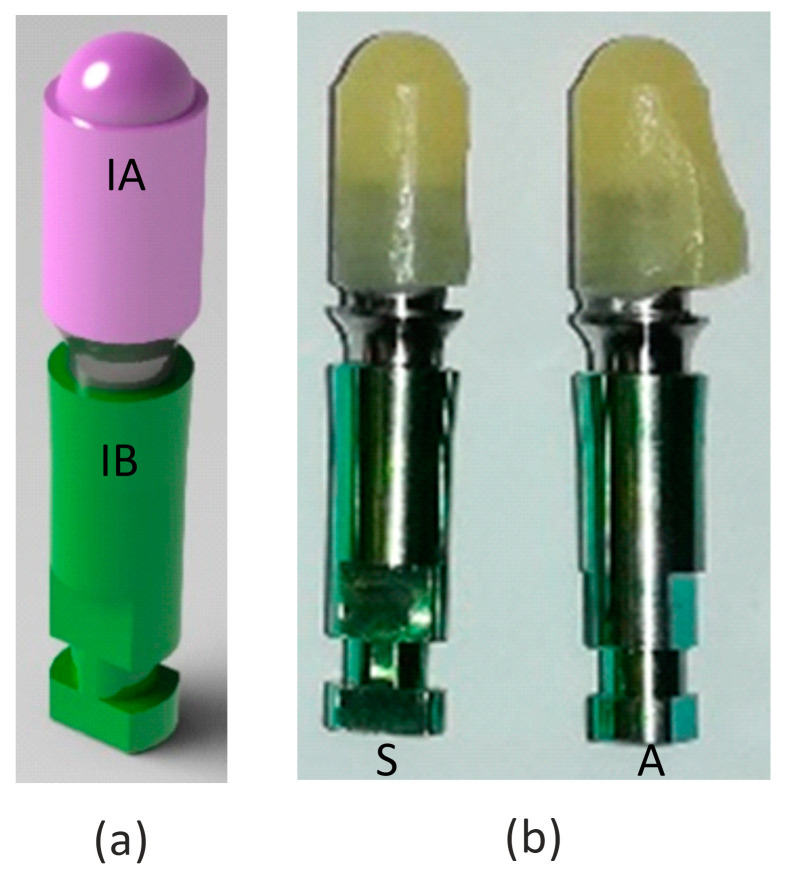
Specimen geometries: (**a**) a 3D model; (**b**) example of the two geometries of a real specimen. In figure: IA, the implant abutment; IB, the implant body; S, symmetric geometry; A, asymmetric geometry.

In the present study, four types of material were analyzed in monotonic static and fatigue tests: lithium disilicate, translucent zirconia, fiber-reinforced PMMA, and ceramic-reinforced PEEK material, namely Type A, Type B, Type C, and Type D (see Table 1), respectively. These materials were used for the fabrication of the implant abutment, IA, cemented to the implant body, IB, as shown in the rendering of the specimen’s 3D model of Figure 2a), and used for the design of the loading setup.

The geometry of the implant body is not that of a typical threaded rod used to tighten an implant to a patient’s jawbone. Instead, the implant body was shaped as a rod, with a small conical angle (see Figure 2a) that allowed the specimen to be clamped to the specimen holder, with a low pressure being manually applied before the test. Thanks to the mechanical solution adopted for the loading setup, the compressive load of the tests does not imply that any disassembling or releasing effects occurred.

To investigate whether the mechanical behavior of the abutment was affected by its shape, two different geometries were tested for all four materials. The specimens made out of the four materials are shown below with symmetrical (S) and asymmetrical (A) geometries, in Figure 2b. The four materials can be easily distinguished from one another by their color and surface texture; therefore, blinding was not possible during the tests.

### 2.3. Testing Protocols

The first mechanical test performed on the specimens was a monotonic static test, which defined the static strength of every combination of material and geometry. The tests were carried out under the displacement control mode at a crosshead speed of 0.1 mm/min, and five different samples were tested for each sample type. Due to considerable statistical dispersion in the experimental results caused by variations in material properties and manufacturing processes (such as non-standardized cementation), the static strength was determined as the average of five valid tests. A single test was considered valid when a failure occurred: (i) in the implant abutment; (ii) at a significant load; (iii) without a clear variation in the load setup geometry due to abnormal deformation of the specimen before failure.

Following the identification of static strength and the potential influence of the specimen’s geometry, static strength was used as reference value for subsequent fatigue tests. More specifically, every material was subjected to fatigue tests using at least four load levels, with the test being repeated at each of the load levels at least three times. Load controlled tests were carried out with a load ratio R (F_min_/F_max_) equal to 0.1, as prescribed by the ISO standard. The load frequency was set to 10 Hz, which is less than 15 Hz, as specified in the standard in case of dry tests, and with a runout reference value equal to 5 × 10^6^ cycles, corresponding to five years of clinical service [17,21]. The peak value of load levels was initially set to 80% of the static strength and reduced accordingly in subsequent load levels.

## 3. Results

### 3.1. Static Strength

The results of the static tests for all tested samples are summarized in Table 2, together with significant statistical parameters. Student’s two-sample location t-test was applied to two groups of five measurements to find the potential influence of the two geometries considered on static strength for all four types of materials. A null hypothesis assumed that the means of the two populations were equal. The threshold *p*-value was equal to 0.05. No significant variation between the two sample geometries was captured for material Types A, B, and D, whereas a large variation between the two geometries of the Type C material was revealed, which is fiber-reinforced polymer. These results emphasizes the significant coupling between material and geometry in fiber-reinforced polymer. Specifically, the alignment of fibers is dependent on the geometry and can generate anisotropic responses in the sample. As a result, static strength was determined by averaging the measurements for Types A, B, and D, without distinguishing between symmetric and asymmetric geometries. However, for Type C, the two geometries were treated as distinct sample types.

**Figure 3:**
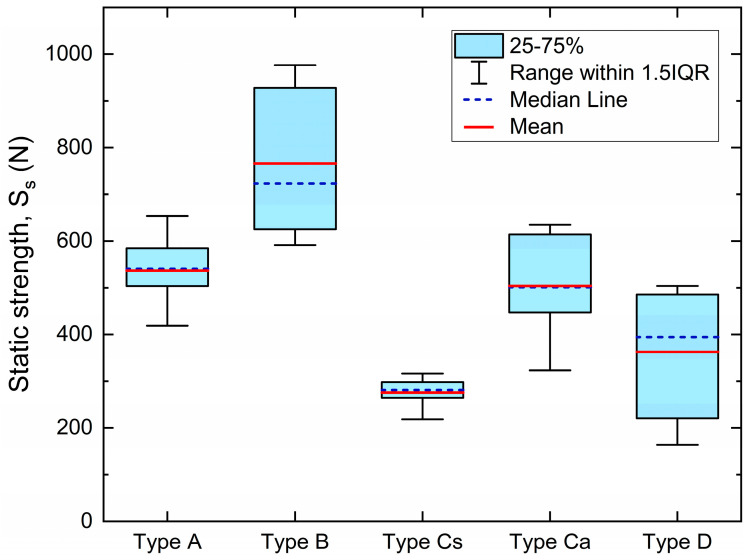
Results of static tests for all sample types.

For the sake of comparison, strength data are also plotted in box charts in Figure 3, which report mean and median values, the 1.5 interquartile range, and the 25–75% percentile range.

### 3.2. Fatigue Strength

The findings from the static tests were utilized to develop the fatigue experimental campaign. Specifically, for Types A, B, and D, no distinctions were made between symmetric and asymmetric geometries. However, for Type C, two distinct sample geometries were examined: CS and CA for symmetric and asymmetric geometries, respectively. The measured static strengths (see Table 2) were also used as reference values to establish load levels for fatigue experiments.

**Figure 4:**
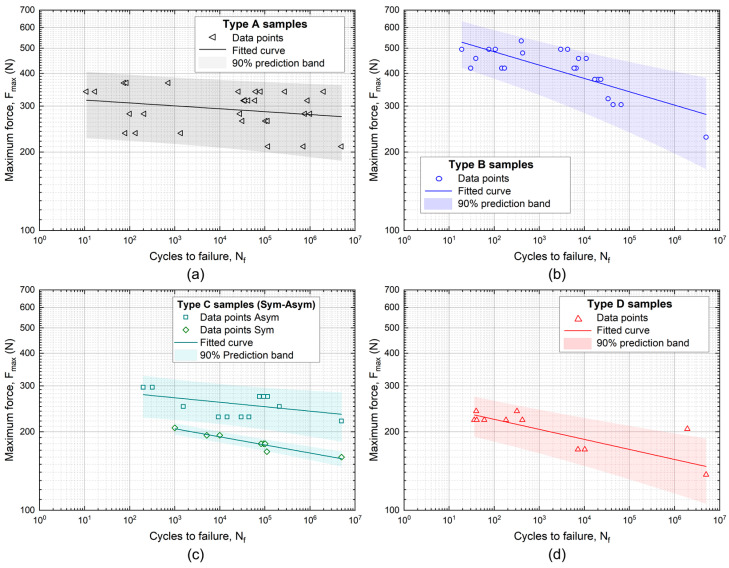
Fatigue data for the different sample types: (**a**) Type A; (**b**) Type B; (**c**) Type C, symmetrical and asymmetrical; (**d**) Type D.

Log–log diagrams in Figure 4 show the results of fatigue experiments for all the samples’ types. The graphs show data points and corresponding fitted curves, together with prediction bands with 90% confidence levels, obtained by the following power law relation typically used to model and predict the fatigue behavior of a material:

(1) $$P_{f}=a\;N_{f}{}^{b}$$

with *P_f_* being fatigue strength (that in the case of the present study is a force), *N_f_* being the number of cycles to failure, and coefficient *a* and exponent *b* being the two constants evaluated in the aforementioned fitting procedure.

According to the usual procedure applied to determine the fatigue strength of a material, at least three valid tests per load level and at least four load levels per sample type were carried out. The evaluation of the power law parameters (*a* and *b*) of Equation (1) were calculated by performing least square fitting on the fatigue strength values.

The fatigue experiment results are in agreement with the overall results obtained from the static tests, which state that Type A and Type B samples showed a better fatigue performance than Type C and Type D did. Furthermore Figure 4c confirms the clear different strength for Type C and also in the case of fatigue loading conditions for symmetrical and asymmetrical geometries by showing totally non-intersecting prediction bands. This large difference can be attributed to the composite structure of Type C, which could generate material anisotropies with possibly significantly different mechanical behaviors due to the two sample geometries.

**Figure 5:**
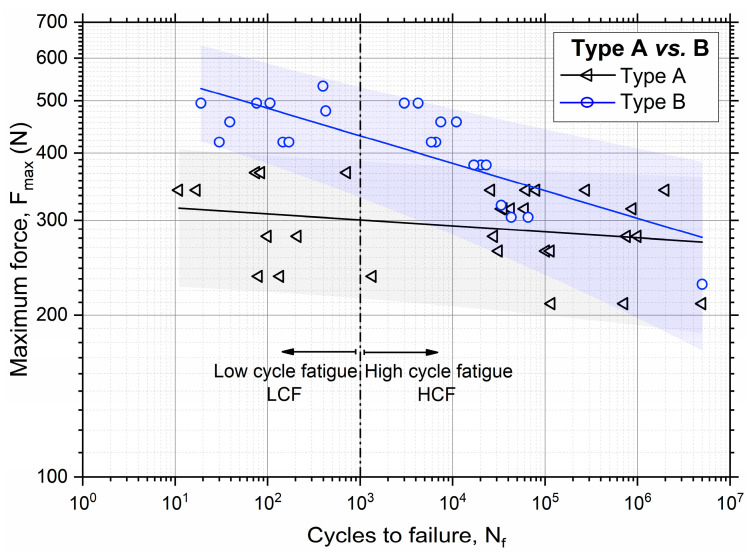
Comparison of the fatigue data and prediction bands for Type A and Type B samples, with evidence of low cycle fatigue (LCF) and high cycle fatigue (HCF) regimes.

Figure 5 shows a direct comparison between the Type A and Type B samples, which were made of the best performing materials among the four candidates. The graphs in Figure 5 show that the B samples had a higher fatigue strength with respect to that of Type A, according to their superior static strength (about 766 N vs. 528 N). This is clearly evident in the low cycle fatigue (LCF, N_f_ < 10^3^) regime, with a fatigue strength at N_f_ = 10^3^, namely $S_{f1k}$, of about 430 N for Type B and 300 N for Type A. However, the differences between the two materials tend to vanish in the high cycle fatigue (HCF) regime, with the complete overlapping of prediction bands (*p* = 90%) at the runout (5 × 10^6^ cycles). In particular, the two materials show nearly the same fatigue strength, namely, $S_{f5M}$, which is around 280 N. In addition, the Type B samples show a slightly lower strength at *p* = 90% with respect to that of Type A, which is a direct consequence of the higher slope of the fitting curve and larger data scatter.

**Figure 6:**
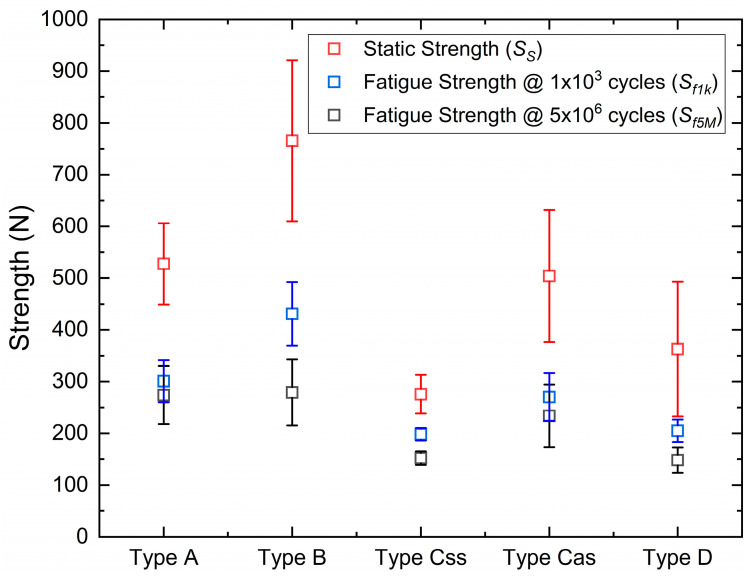
Static strength ($S_{S}$) and fatigue strength at $N_{f}=10^{3}$ ($S_{f1k}$ ) and $N_{f}=5\times 10^{6}$ $S_{f5M}$ for all sample types.

Figure 6 shows a comparison between static strength ($S_{S}$) and fatigue strength at 1 × 10^3^ and 5 × 10^6^ cycles ($S_{f1k}$ $S_{f5M}$), as obtained from data fitting and the statistical analysis of all sample types.

Table 3 summarizes the parameters of the power law prediction curves of Equation (1) in terms of coefficient *a* and exponent *b*, together with the predicted endurance limit *S_f5M_* for all the sample types.

**Figure 7:**
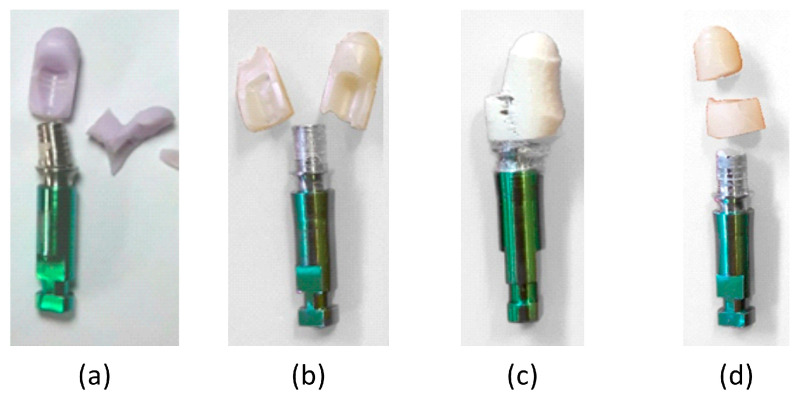
Typical failure modes of the tested materials: (**a**) Type A; (**b**) Type B; (**c**) Type C; (**d**) Type D.

Finally, Figure 7 shows the failure mode of the four types of materials. Types A, B, and D exhibited quite similar failures, with longitudinal or transversely fractured surfaces, which are typical of brittle behavior, whereas Type C showed a failure mode typical of fibrous composite materials, with the gradual formation of a fractured surface, and the phenomenon of fiber bridging occurred.

## 4. Discussion

In the current study, the static and fatigue strength values were assessed in terms of force rather than stress, which is the conventional approach in the mechanical analysis of materials. The rationale behind this lies in the intricate geometry of the sample, specifically, the abutment, which necessitates prior investigation to determine the stress state resulting from the applied load and the geometry. This approach is additionally justified when we consider that all abutments have the same dimensions and shape, except for symmetrical and asymmetrical geometries, which have been duly considered. The objective of this work was to conduct a comparative study and mechanically characterize new materials that are potential candidates for dental implantology. The obtained results agree with those of previous studies [19,22,23]. Sivrikaya et al. found that zirconia abutments showed a lower stress distribution compared to that of titanium abutments, indicating that zirconia abutments may be a better choice for dental implants in patients who exhibit parafunctional habits. Furthermore, Sarot et al. demonstrated that the CFR-PEEK implants showed lower stress levels compared to those of traditional titanium implants, indicating that CFR-PEEK may be a viable alternative material for dental implants, while it was mentioned that abutments made of titanium or machinable precious alloy exhibited similar mechanical properties and fracture resistance. However, machinable precious alloy abutments showed higher corrosion resistance and better aesthetic results compared to those of titanium abutments [24]. However, it is important to take into consideration that the manufacturing technique used for fiber-reinforced polymers has a significant impact on the final characteristics of the devices produced. The study revealed that the final properties of the restoration depended on the manufacturing techniques and the operator’s experience The results of the study provide useful information for clinicians to select the appropriate material for customized abutments, depending on their specific requirements and the patient’s needs. The study suggests that further analysis is necessary to determine the most suitable material to be used, considering the feasibility of its manufacture, in vitro biomechanical behavior, and its biological safety.

## 5. Conclusions

The mechanical performance of four different types of commercial abutment materials, including ceramics and polymer composites, was investigated for both static and fatigue behaviors in accordance with ISO standard 14801:2016. Two different material geometries were also analyzed. The main results are summarized as follows:

− In comparison to polymer-based composites (PMMA and PEEK), ceramics (disilicate and zirconia) demonstrated significantly more static strength, with mean values exceeding 500 N for disilicate and 750 N for zirconia.
− The results of the fatigue tests confirmed the trend observed for static strength, with disilicate and zirconia exhibiting the highest endurance limit values at 5 × 10^6^ cycles, with mean values of around 280 N for both materials.
− The power law fatigue parameters obtained for all materials provided the fatigue strength for a wide range of life cycles and highlighted the sensitivity of each material to fatigue loading conditions.
− For nominally isotropic materials, such as ceramics and PEEK, no geometric effects were observed. However, significant material–geometry coupling was noted for fiber-reinforced PMMA in terms of both static and fatigue strength.

## Figures and Tables

**Table 1:** Tested materials.

Material ID	Material Type	Trade Name/Manufacturer	Manufacturing Method
Type A	Lithium disilicate	IPS Emax-Ivoclar	Sintering
Type B	Translucent zirconia	Katana, Kouraray-Noritake	Sintering
Type C	Fiber-reinforced PMMA	Bre.CAM, Multilayer, Bredent	Milling
Type D	Ceramic-reinforced PEEK	breCAM.BioHPP, Bredent	Milling

**Table 2:** Static strength measurements (in Newtons) and the statistical properties obtained from the four analyzed materials. S and A refer to symmetric and asymmetric geometries, respectively; $\bar{S_{S}}$ and ***σ*** are the mean value and the standard deviation of the static strength of the samples, respectively.

		Type A		Type B		Type C		Type D	
		S	A	S	A	S	A	S	A
Sample ID	1	540.3	446.4	654.9	965.5	218.5	323.1	163.9	175.6
	2	514.5	503.7	591.3	595.6	298.3	614.2	220.7	504.0
	3	434.8	574.4	741.6	625.1	316.2	501.2	496.6	403.1
	4	584.5	653.4	872.6	976.5	264.6	447.0	377.9	414.0
	5	418.5	605.6	704.4	927.9	281.1	634.7	385.4	485.5
Sample	$\bar{S_{S}}$	498.5	556.7	713.0	818.1	275.7	504.0	328.9	396.4
	σ	70.5	82.2	105.5	190.8	37.3	127.7	134.8	131.0
*p*-value		0.264		0.321		0.014		0.445	
Material	$\bar{S_{S}}$	527.6		765.5		N/A		362.7	
	σ	78.4		155.6		N/A		130.2	

**Table 3:** Parameters of the power law prediction curves in terms of coefficient a and exponent b and predicted endurance limit Sf5M for all sample types.

		Type A	Type B	Type C_S_	Type C_A_	Type D
Power law parameters	*a* (N)	324.6 ± 26.2	613.0 ± 45.0	245.5 ± 9.8	303.8 ± 30.8	266.5 ± 15.3
	*b*	−0.011 ± 0.008	−0.051 ± 0.010	−0.031 ± 0.003	−0.017 ± 0.010	−0.038 ± 0.007
Fatigue strength	*S_f1k_* (N)	300.8 ± 42.7	431.0 ± 64.7	198.2 ± 12.2	270.1 ± 46.1	205.0 ± 21.7
	*S_f5M_* (N)	273.9 ± 56.1	279.1 ± 64.0	152.2 ± 13.2	233.7 ± 60.2	148.3 ± 24.6

## Data Availability

Not applicable.
